# Supplementary material for: Acoustic monitoring reveals a diel rhythm of an arctic seabird colony (little auk, Alle alle)
Source: Commun Biol. 2024 Mar 15;7:307. doi: 10.1038/s42003-024-05954-8 (PMC10942998; doi:10.1038/s42003-024-05954-8)
Supplement: Supplementary file 2 — Supplementary Information [file 42003_2024_5954_MOESM2_ESM.pdf]

Supplementary Information

Supplementary Note 1 Conflicting interpretations of the diel rhythm.

Evans et al. [34] counted little auks on land and sea and found that colony attendance changed from the incubation (11–20 July 1974) to late nestling (31 July – 10 August 1974) period. Specifically, during incubation, the number of birds reached a maximum between 06:00 and 12:00. During the late nestling period, the colony had the largest number of birds at around 02:00 and was almost deserted between 15:00 and 19:00, similar to our pattern and the impressions of Ferdinand [1]. Report by Lyngs et al. [35] from Northwest Greenland also suggests that before the fledging, the highest occurrence of adult birds recorded by a camera fell between 12:00 and 15:00, and shifted to early morning when fledging started (our Supplementary Fig. 3 reveals a similar tendency). Furthermore, Evans et al. [34] reported that the number of feeds to nestling chicks reached a maximum around 02:00 and a minimum around 14:00, and that this feeding rhythm closely resembled the vertical migration of the copepod *Calanus finmachicus* – this key prey species for the little auk becomes available close to the water surface after midnight and moves deeper at around 14:00. Evans et al. and Ferdinand [1,34] also observed that the little auks slept very little on land, further suggesting that the birds remain socially active while in the colony.

A census of the attendance of the little auks was conducted at a breeding ground in Svalbard throughout the different stages of the breeding season (13 May – 20 August 1980; 30 July – August 27, 1983 [36]); it was supplemented with a census of glaucous gulls, *Larus hyperboreus*, the key predator of the little auk in Svalbard. During the late incubation period, peak attendance was observed at noon and shifted in the later stages closer to midnight, consistent with the observations of Evans et al. [34], Lyngs et al. [35], and our data from Qoororsuaq (Supplementary Fig. 3). Interestingly, from the period before egg-laying to the period of incubation the little auk attendance cycles became shorter and persisted until the end of the season (from ~48 to ~24 h) [36].

The number of glaucous gulls reflected the rhythm of the little auks' attendance but was less noticeable during the chick-nesting period [36]. In general, the number of gulls increases as the wind speed increases because it improves their hunting efficiency, and the little auks may then prefer to stay at sea because they can dive to escape predation. Nevertheless, Stempniewicz [36] argued "that the attendance rhythm of Little Auks imposes the activity rhythm on the gulls and not vice versa". He suggested that the factor that really determines the attendance rhythm of the little auk is food availability and agrees with Evans et al. [34] that *Calanus finmachicus* is the main food of the little auk and has a clear diel rhythm at high latitudes. Lyngs et al. [35] conducted a 24-h watch to record the number of fly-outs of little auks (3 August 2010): they also observed that the

number of fly-outs approximately followed the attendance, with 63% of the fly-outs caused by glaucous gulls.

A recent study in Svalbard during the chick-rearing period (27 July – 7 August, 2009; 23 July – 8 August 2010; Wojczulanis-Jakubas et al. [37]) also detected a semi-diel rhythm in the little auk colony (an average cycle of 23.2 h; range from 19.9 to 30.8 h), with maximum attendance after midnight and minimum attendance late in the afternoon. This pattern was nearly identical to the previous end-of-July data (e.g., see 30 July 1980 in fig. 1d by Stempniewicz [36]). The aforementioned deviation from the 24-h cycle was detected from 48-h-long data [37], but nevertheless is intriguing and could be further investigated, as it might correspond to free-running patterns under continuous polar light [17].

In contrast to Evans et al. [34], the study by Wojczulanis-Jakubas et al. [37] detected no diurnal pattern in chick feeding and also dismissed the diel vertical migration of zooplankton under the midnight sun as a weak and unlikely potential driver. Here, we note that the number of feeds was estimated differently: Evans et al. [34] calculated the number of feeds (per chick) by measuring the weight change of the chicks (n = 18) every 2 h, after confirming the reliability of the method using a 24-h continuous concealed watch. However, Wojczulanis-Jakubas et al. [37] visually counted birds with full gular poaches arriving in the colony. Moreover, they [37] cited a different species of zooplankton as the key prey of the little auk *Calanus glacialis*. Nevertheless, Wojczulanis-Jakubas et al. [37] proposed that the ultimate cause of the diel attendance rhythm was predation pressure. Specifically, the authors suggested that increased shadowing over the area and the low-light conditions favored the detection of danger from the glaucous gull so that socializing in low sun was safer.

Supplementary Note 2 Predators in Siorapaluk?

Roby et al. [24] observed glaucous gulls, common ravens, and arctic foxes (*Alopex lagopus*) in Siorapaluk. They noted a pronounced behavioral effect of gulls (and ravens) on the little auk colonies at Cape Atholl ("If passing gulls were frequent, the nesting slope was virtually devoid of birds for over half an hour"), but that "Foxes were tolerated at a distance of 2 m without causing panic flights." At the end of July 2016, we also saw gulls and, presumably, common ravens at the colony near Siorapaluk, but without any prolonged behavioral response from the little auks (Supplementary Fig. 4). To the south of Siorapaluk, Lyngs et al. [35] observed up to 9.5 fly-outs of little auks per hour, which were primarily caused by glaucous gulls (63%) and foxes (4%).

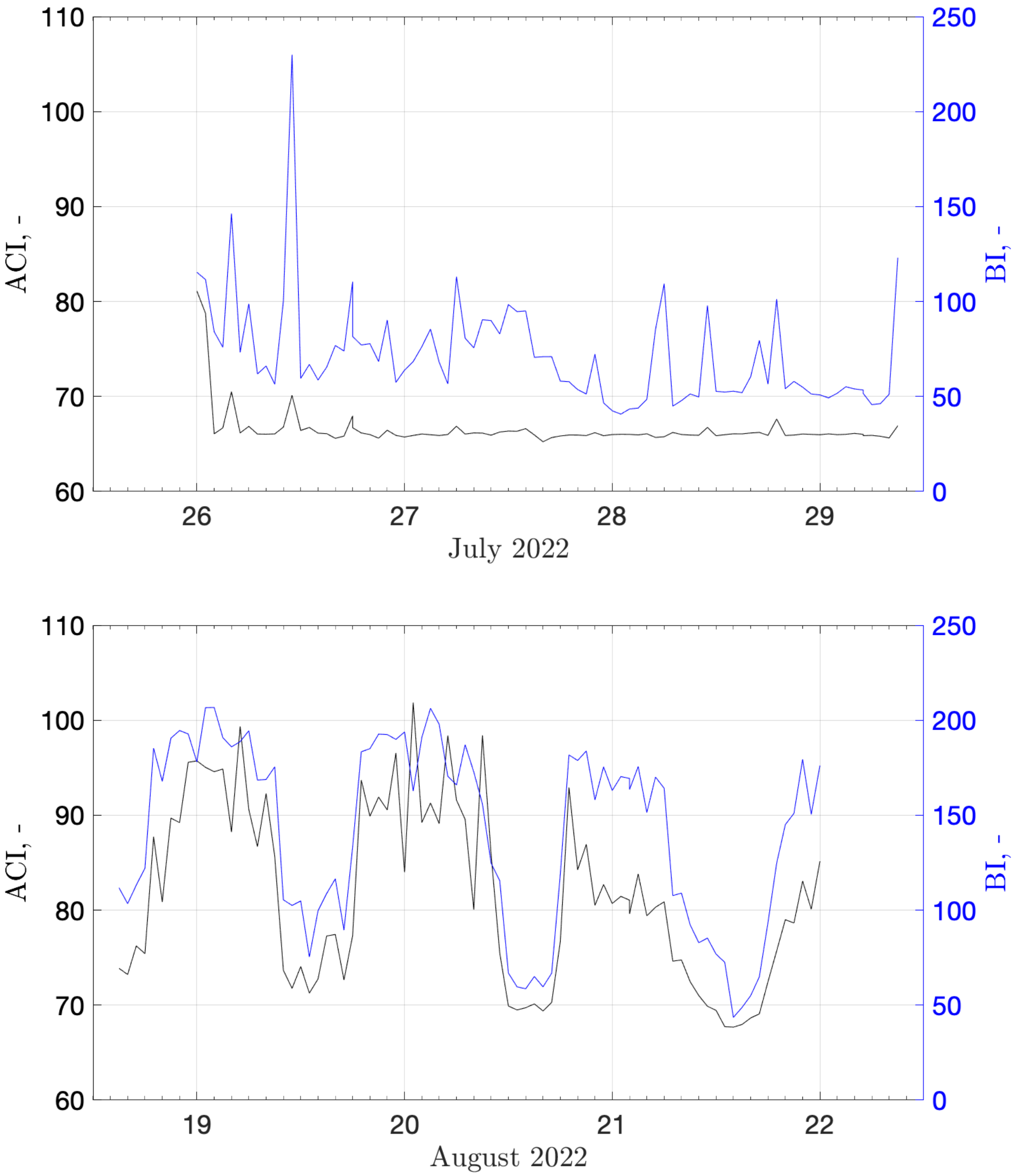

Supplementary Fig. 1. Biophony scores. The Acoustic Complexity and Bioacoustic indices for July and August data (hourly). The Bioacoustic Index anomaly around 11:00 on 26 July 2022, is due to a broadband signal of a helicopter.

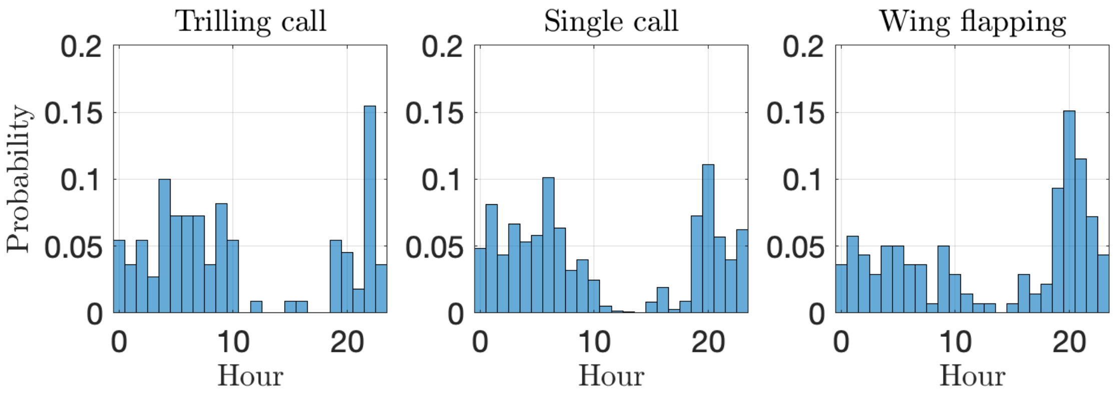

Supplementary Fig. 2. Little auk colony sounds by the time of day. Diurnal variation in relative numbers of different sound types detected in the little auk colony (18–22 August 2022).

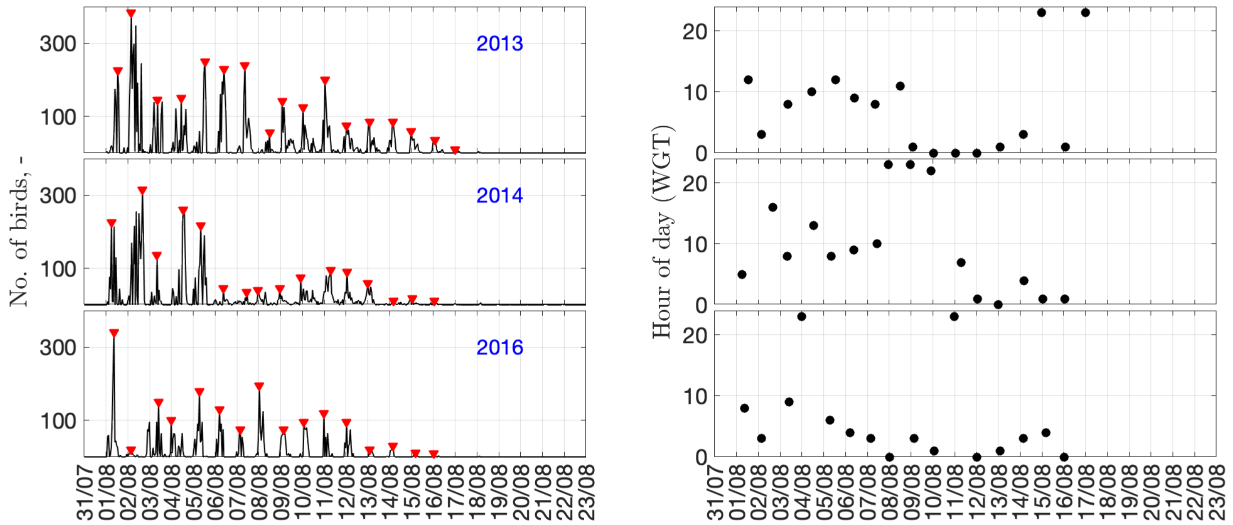

Supplementary Fig. 3. Little-auk colony attendance in Qoororsuaq (2013, 2014, 2016). Number of sitting birds recorded by camera in Qoororsuaq, Northwest Greenland, between 31 July and 1 September 2013, 2014, and 2016 (the x-axis is truncated on 23 August because birds had left). Red triangles show the daily peak, the hour of occurrence of which is provided on the right subplot (peaks lower than 10 were ignored). Source data are available as Supplementary Data 1).

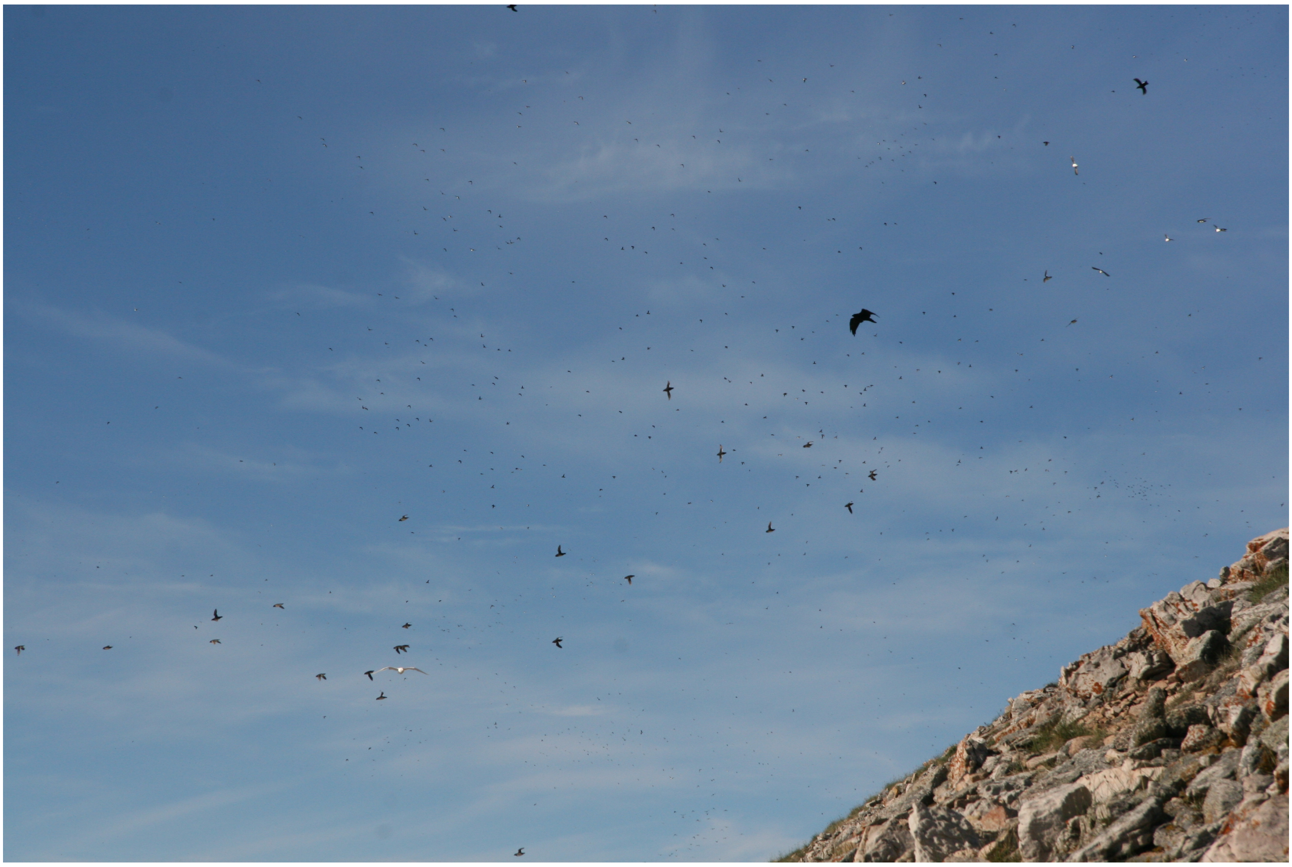

Supplementary Fig. 4. Predators. Gull and a raven flying through the little auk colony near Siorapaluk, 31 July 2016 (Photo: E.A. Podolskiy).
