# Supplementary material for: Acoustic monitoring reveals a diel rhythm of an arctic seabird colony (little auk, Alle alle)
Source: Commun Biol. 2024 Mar 15;7:307. doi: 10.1038/s42003-024-05954-8 (PMC10942998; doi:10.1038/s42003-024-05954-8)
Supplement: Supplementary file 3 — Description of Additional Supplementary Files [file 42003_2024_5954_MOESM3_ESM.pdf]

Description of Additional Supplementary Files

File name: Supplementary Audio 1

Description: Night noise of the little auk colony.

File name: Supplementary Audio 2

Description: Day noise of the little auk colony.

File name: Supplementary Data 1

Description: Little auk counts recorded by camera in Qoororsuaq (August 2013, 2014, 2016).

File name: Supplementary Data 2

Description: Biorhythms-related values from little auk colonies in other regions (source data for Fig. 4a).
