# Supplementary material for: Acoustic monitoring reveals a diel rhythm of an arctic seabird colony (little auk, Alle alle)
Source: Commun Biol. 2024 Mar 15;7:307. doi: 10.1038/s42003-024-05954-8 (PMC10942998; doi:10.1038/s42003-024-05954-8)
Supplement: Supplementary file 5 — Supplementary Data 2 [file 42003_2024_5954_MOESM5_ESM.docx]

**Supplementary Data 2**

**Evans, 1981**

**the number of birds**

Hour = [0,2.3,4.3,6.5,9.0,10.1,12.3,14.5,16.6,18.4,20.2,21.3,21.9];

Nb = [130.6,140.7,102.1,123.9,101.3,96.3,63.6,31.0,19.7,10.0,41.4,88.3,115.5];

**Evans, 1981**

**the number of feeds to chicks**

Hour = [0, 1.7, 3.5, 5.3, 6.9, 9.6, 11.6, 14.0, 16.1, 18.4, 19.5, 20.4, 23.1, 24.0];

Nf =[12.0, 14.9, 11.7, 11.7, 10.8, 5.4, 3.3, 1.6, 2.0, 3.3, 7.0, 9.7, 12.2, 11.9];

**Wojczulanis-Jakubas et al., 2020**

**the number of birds**

Hour = [0.0,0.9,1.9,3.0,4.0,5.0,6.0,7.0,8.0,9.0,10.0,11.0,12.0,13.0,14.0,15.0,16.0,17.0,18.0,19.0,20.0,21.0,22.0,23.0];

Nbw = [28.9,29.5,29.7,29.1,28.3,26.9,25.1,22.3,19.7,16.9,13.8,11.2,9.6,8.2,7.2,6.7,6.7,7.3,8.5,10.2,12.5,15.1,18.5,22.2];

**The corresponding 95% confidence limits, Nbwu**

Hour = [25.3,26.8,27.6,27.5,26.4,25.0,23.0,20.5,17.6,14.8,11.6,9.1,7.4,6.1,5.1,4.6,4.7,5.4,6.7,8.4,10.5,13.1,15.8,18.6];

Nbwu=[32.3,32.1,31.6,30.9,29.9,28.7,26.8,24.4,21.6,18.7,15.4,13.0,11.1,10.0,9.0,8.5,8.4,9.1,10.1,11.7,14.1,17.0,20.9,25.6];

**Evans, 1981**

**zooplankton abundance, Nz**

Hour = [0, 3.1, 6.2,7.6,8.9,9.8,10.7,11.6,12.3,12.5,12.9,13.4,14.0,14.7,15.4,16.1,16.6,17.3,17.8,18.2,18.3,18.5,18.8,19.0,21.1,22.9,24.0];

Nz = [1317.2,1312.5,1310.2,1021.9,766.4,574.2,379.7,213.3,60.9,49.2,72.7,96.1,105.5,110.2,114.8,93.8,77.3,51.6,32.8,18.8,150.0,351.6,569.5,754.7,649.2,1031.3,1286.7];

**Lyngs et al., 2010**

**the number of chicks out**

Hour = [0:1:2,3,4,5,6,7,8,9,10,11,12,13,14,15,16,17,18,19,20,21,22,23];

Nc = [1,4,4,8,5,7,7,5,6,3,1,0,4,1,1,0,3,0,0,1,0,0,1,0];
