# Supplementary material for: Acoustic monitoring reveals a diel rhythm of an arctic seabird colony (little auk, Alle alle)
Source: Commun Biol. 2024 Mar 15;7:307. doi: 10.1038/s42003-024-05954-8 (PMC10942998; doi:10.1038/s42003-024-05954-8)
Supplement: Supplementary file 8 — Reporting Summary [file 42003_2024_5954_MOESM8_ESM.pdf]

nature portfolio

Corresponding author(s): Evgeny Podolskiy

Last updated by author(s): Dec 6, 2022

Reporting Summary

Nature Portfolio wishes to improve the reproducibility of the work that we publish. This form provides structure for consistency and transparency in reporting. For further information on Nature Portfolio policies, see our Editorial Policies and the Editorial Policy Checklist.

Statistics

For all statistical analyses, confirm that the following items are present in the figure legend, table legend, main text, or Methods section.

n/a	Confirmed	
☒	☐	The exact sample size (*n*) for each experimental group/condition, given as a discrete number and unit of measurement
☒	☐	A statement on whether measurements were taken from distinct samples or whether the same sample was measured repeatedly
☒	☐	The statistical test(s) used AND whether they are one- or two-sided<br>*Only common tests should be described solely by name; describe more complex techniques in the Methods section.*
☒	☐	A description of all covariates tested
☒	☐	A description of any assumptions or corrections, such as tests of normality and adjustment for multiple comparisons
☐	☒	A full description of the statistical parameters including central tendency (e.g. means) or other basic estimates (e.g. regression coefficient) AND variation (e.g. standard deviation) or associated estimates of uncertainty (e.g. confidence intervals)
☒	☐	For null hypothesis testing, the test statistic (e.g. *F*, *t*, *r*) with confidence intervals, effect sizes, degrees of freedom and *P* value noted<br>*Give P values as exact values whenever suitable.*
☒	☐	For Bayesian analysis, information on the choice of priors and Markov chain Monte Carlo settings
☒	☐	For hierarchical and complex designs, identification of the appropriate level for tests and full reporting of outcomes
☒	☐	Estimates of effect sizes (e.g. Cohen's *d*, Pearson's *r*), indicating how they were calculated

Our web collection on statistics for biologists contains articles on many of the points above.

Software and code

Policy information about availability of computer code

Data collection	n/a
Data analysis	Matlab R2018b

For manuscripts utilizing custom algorithms or software that are central to the research but not yet described in published literature, software must be made available to editors and reviewers. We strongly encourage code deposition in a community repository (e.g. GitHub). See the Nature Portfolio guidelines for submitting code & software for further information.

Data

Policy information about availability of data

All manuscripts must include a data availability statement. This statement should provide the following information, where applicable:

- Accession codes, unique identifiers, or web links for publicly available datasets
- A description of any restrictions on data availability
- For clinical datasets or third party data, please ensure that the statement adheres to our policy

Acoustic data are publicly available through Zenodo data repository: doi:10.5281/zenodo.7302925 & 2022;doi:10.5281/zenodo.7306102.

Human research participants

Policy information about studies involving human research participants and Sex and Gender in Research.

Reporting on sex and gender	n/a
Population characteristics	n/a
Recruitment	n/a
Ethics oversight	n/a

Note that full information on the approval of the study protocol must also be provided in the manuscript.

Field-specific reporting

Please select the one below that is the best fit for your research. If you are not sure, read the appropriate sections before making your selection.

☐ Life sciences ☐ Behavioural & social sciences ☒ Ecological, evolutionary & environmental sciences

For a reference copy of the document with all sections, see nature.com/documents/nr-reporting-summary-flat.pdf

Ecological, evolutionary & environmental sciences study design

All studies must disclose on these points even when the disclosure is negative.

Study description	acoustic monitoring
Research sample	n/a
Sampling strategy	n/a
Data collection	Data was collected in situ in summer 2022, by deployment of acoustic recorders under rocks for protection from the wind.
Timing and spatial scale	26-29 July 2022, 18-22 August 2022; no monitoring was planned between July 29 and August 18 due to logistical constrains of the field campaign and battery life of a recorder. The spacial scale between two sites was about 3 km. Acoustic records were taken with a frequency of 44,100 Hz, which allows to fully cover the audible range of sounds.
Data exclusions	no data was excluded from the analysis.
Reproducibility	multi-day measurements confirmed the existence of the acoustic pattern.
Randomization	n/a
Blinding	n/a

Did the study involve field work? ☒ Yes ☐ No

Field work, collection and transport

Field conditions	Clear and moderately mild weather, with no strong wind dominated the study period.
Location	Siorapaluk (77◦47’ N 70◦38’ W), Northwest Greenland
Access & import/export	Noninvasive studies near Siorapaluk do not require any special permissions from the Government of Greenland with which we closely work for other research topics. Furthermore, no samples were imported or imported, and no dual-use instrumentation has been involved into our setup.
Disturbance	Observations were noninvasive and autonomous. Therefore, no disturbance was made to the birds. Only for sensor deployment and collection, we approached the colony, and these several marginal minutes are of negligible disturbance as we know from personal observations and previous literature (Roby et al., 1981).

Reporting for specific materials, systems and methods

We require information from authors about some types of materials, experimental systems and methods used in many studies. Here, indicate whether each material, system or method listed is relevant to your study. If you are not sure if a list item applies to your research, read the appropriate section before selecting a response.

Materials & experimental systems

n/a	Involved in the study
☒	☐ Antibodies
☒	☐ Eukaryotic cell lines
☒	☐ Palaeontology and archaeology
☒	☐ Animals and other organisms
☒	☐ Clinical data
☒	☐ Dual use research of concern

Methods

n/a	Involved in the study
☒	☐ ChIP-seq
☒	☐ Flow cytometry
☒	☐ MRI-based neuroimaging
